# Predictive Value of 1-Hour Glucose Elevations during Oral Glucose Tolerance Testing for Cystic Fibrosis-Related Diabetes

**DOI:** 10.1155/2023/4395556

**Published:** 2023-04-17

**Authors:** Andrea N. Lorenz, Laura Pyle, Joon Ha, Arthur Sherman, Melanie Cree-Green, Scott D. Sagel, Kristen J. Nadeau, Christine L. Chan

**Affiliations:** ^1^Pediatric Endocrinology, Department of Pediatrics, Children's Hospital Colorado, University of Colorado Anschutz Medical Campus, Aurora, CO 80045, USA; ^2^Department of Biostatistics, University of Colorado Anschutz Medical Campus, Aurora, CO 80045, USA; ^3^Laboratory of Biological Modeling, National Institutes of Health, Bethesda, MD, USA; ^4^Breathing Institute, Department of Pediatrics, University of Colorado Anschutz Medical Campus, Aurora, CO 80045, USA

## Abstract

**Background:**

In cystic fibrosis-related diabetes (CFRD) screening, oral glucose tolerance test (OGTT) thresholds for detecting prediabetes and diabetes are defined by the 2-hour glucose (2 hG). Intermediate OGTT glucoses, between 0 and 2 hours, that are ≥200 mg/dL are deemed “indeterminate,” although lower 1-hour glucose (1 hG) thresholds identify those at increased risk of type 2 diabetes in other populations, and may also better predict clinical decline in CF. Studies of 1 hG thresholds <200 mg/dL in people with CF are limited.

**Methods:**

A single center, retrospective chart review was performed of patients with 1 hG available on OGTTs collected between 2010 and 2019. In patients with ≥2 OGTTs, Kaplan–Meier analysis estimated likelihood of progression to CFRD based on a high vs. low 1 hG. In patients with ≥1 OGTT, mixed-effects models tested whether baseline 1 hG and 2 hG predicted growth and lung function trajectories.

**Results:**

A total of 243 individuals with CF were identified with at least 1 OGTT including a 1 hG, and *n* = 177 had ≥2 OGTTs. Baseline age (mean ± SD) was 12.4 ± 2.6 years with 3.2 ± 1.4 years of follow-up. Twenty-eight developed CFRD. All who developed CFRD had a 1 hG ≥ 155 mg/dL prior to 2 hG > 140 mg/dL. The average 1 hG was 267 mg/dL when 2 hG ≥ 200 mg/dL. In a subset with baseline 2 hG < 140 mg/dL, 1 hG ≥ 140 mg/dL conferred an increased 5 years risk of CFRD (*p*=0.036). Baseline 2 hG predicted decline in FEV1%predicted, but 1 hG did not.

**Conclusions:**

In youth with CF, 1 hG ≥ 140 mg/dl is an early indicator of CFRD risk. However, 2 hG, rather than 1 hG, predicted lung function decline.

## 1. Introduction

Outcomes for cystic fibrosis (CF) have improved in recent years, illustrated by a median predicted survival of 59.0 years in 2020 compared to 40 years during the five-year period from 2010 to 2014 [1]. However, increased longevity is anticipated to increase the prevalence of cystic fibrosis-related diabetes (CFRD) and diabetes-related complications [2]. Screening for CFRD is important as even early diabetes increases risk of nutritional and respiratory decline [3]. Furthermore, identification and treatment of CFRD with insulin has been shown to reverse these clinical declines [4–7]. Current screening guidelines recommend annual oral glucose tolerance tests (OGTT), including measurement of fasting and 2-hour glucose (2 hG) concentrations for people with CF (pwCF) starting at 10 years of age [4]. The current cut-offs used for dysglycemia are based on the 2 hG in pwCF and are the same as for other types of diabetes; namely, a 2 hG from 140 to 199 mg/dL is considered prediabetes and ≥200 mg/dL is considered diabetes. A fasting glucose ≥126 mg/dL is also considered consistent with diabetes, although elevations in fasting glucose tend to develop later in the course of diabetes progression in CF [4]. Elevations in intermediate OGTT glucoses ≥200 mg/dL, collected between time 0 and 2 hr, are considered “indeterminate” and have been associated with an increased risk for CFRD [8]. Increasingly, elevations in 1-hour glucose (1 hG) of 155 mg/dL to 200 mg/dL have been described to represent early glucose intolerance, but longitudinal data examining the predictive value of 1 hG, particularly in pediatric populations, are limited. Whether or not lower intermediate cutoffs may be of clinical relevance has not been fully investigated in youth with CF.

We, therefore, examined the predictive value of 1 hG and 2 hG for identifying future CFRD and for detecting clinical decline and examined the relationship between 1 hG and 2 hG, in a retrospective longitudinal analysis of OGTTs obtained at our pediatric CF center over 10 years. We hypothesized that early elevations in 1 hG would be predictive of future CFRD and identify those at greater risk for clinical declines.

## 2. Methods

### 2.1. Study Design and Population

This was a retrospective chart review of electronic health records data from pediatric patients seen at Children's Hospital Colorado's CF Center between 2/2010 and 6/2019. For inclusion in the analysis, patients had to meet the following criteria: absence of CFRD at first OGTT, at least one OGTT between 2/2010 and 6/2019, and a baseline OGTT in this time frame that included the 1 hG. CFRD was defined as an OGTT 2 hG ≥ 200 mg/dL. The study received approval from the Colorado Multiple Institutional Review Board as exempt research under Category 4 Part B as secondary non-human subjects research.

Data collected included age, sex, hemoglobin A1c (A1c), body mass index percentile (BMI %ile), cystic fibrosis transmembrane conductance regulator (CFTR) genotype, pulmonary function testing (PFT), and OGTT results. Glucose and A1c were run at the Children's Hospital Colorado clinical laboratory. Glucose was analyzed by glucose oxidase (Vitros, Ortho Clinical Diagnostics). A1c was measured on a DCA Vantage Analyzer (Siemens), a test method certified by the National Glycohemoglobin Standardization Program. For baseline measures, collected at the time of initial OGTT, data within 90 days of the OGTT visit were included. Genotype was determined to be low risk if at least one allele was a mutation associated with residual CFTR function and high risk if both alleles were minimal function mutations.

Two cohorts were created as follows: Group 1, where all participants were included in analyses of whether baseline 1 hG and/or 2 hG predicts trajectories of BMI %ile and PFTs and Group 2, which included participants with at least two OGTTs in a longitudinal OGTT analysis of whether baseline glucose predicts development of CFRD. The first OGTT with a non-missing 1 hG was used as baseline. A1c and PFT data prior to that date were excluded.

### 2.2. Statistical Analysis

Data were examined for normality and presented as mean ± standard error of the mean for normally distributed data or median (25^th^, 75^th^ percentile) for non-normally distributed data. Unpaired *t*-tests were used to compare patient characteristics if normally distributed and a Welch's *t*-test if not normally distributed. *χ*^2^ was used to evaluate dichotomous parameters.

For the longitudinal cohort of participants with more than one OGTT, Cox proportional hazard models were used to test whether baseline 1 hG or 2 hG predict time to development of CFRD. Time-dependent ROC was used to find the cutoffs of baseline 1 hG and 2 hG that best predicted time to develop CFRD at 1–5 years after baseline. Kaplan–Meier curves were used to estimate the likelihood of progression to CFRD in those with 2 hG above and below 140 mg/dL and in normal glucose tolerance (NGT) participants (i.e., 2 hG < 140 mg/dL and 1 hG < 200 mg/dL) with 1 hG above and below the cut points of 140 mg/dL or 155 mg/dL.

Next, linear mixed effect model fits of 2 hG vs 1 hG were performed using the lme4 package in R [9] to identify the 1 hG values that corresponded to clinically significant 2 hG values.

Finally, mixed-effects models were applied to data from the overall group to test whether baseline glucose predicts trajectories of BMI %ile and PFTs. BMI and PFT measures were assigned to the nearest half-year increment, and time was treated as a categorical variable to allow nonlinear trajectories over time. These models were also adjusted for CFTR modulator use. Modulator use was categorized as highly effective (ivacaftor and elexacaftor/tezacaftor/ivacaftor) or moderately effective (lumacaftor/ivacaftor and tezacaftor/ivacaftor), but the number of visits on highly effective modulators was small and statistical power was too low to adjust for these categories separately.

Level of significance was set at *p*=0.05. All analyses were performed using R (R Core Team, Vienna).

## 3. Results

**Figure 1:**
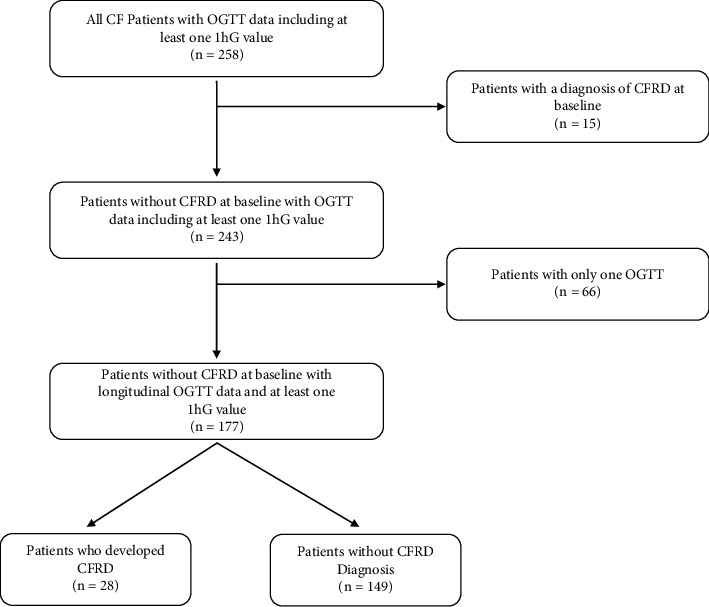
CF cystic fibrosis, OGTT oral glucose tolerance test, CFRD cystic fibrosis-related diabetes, and 1 hG one-hour glucose.

Two hundred and fifty-eight patients with at least one OGTT including a 1 hG were identified (Figure 1). For Group 1, fifteen patients with CFRD at baseline were excluded, leaving 243 patients with a baseline OGTT including 1 hG. The average length of follow up in this overall group was 3.2 ± 1.4 years. For Group 2, one hundred and seventy-seven patients with more than one OGTT were identified with a mean of 3.2 years (range 0.6–7.3 years) of follow-up, 28 of whom developed CFRD in the time frame studied (Table 1). All available follow up OGTTs were included in the analyses for Group 2. In total, 662 fasting and 2 hG and 620 1 hG values were analyzed. There were no significant differences in the number of males vs. females included, and age was similar between sexes (males (*N* = 94, age 12.7 years at initial OGTT), females (*N* = 83, age 12.1 years at initial OGTT)). When evaluating baseline characteristics (Table 2), there were no statistically significant differences between those who developed CFRD during the follow-up window and those who did not. Although those who developed CFRD tended to have higher baseline 1 hG and 2 hG concentrations, the differences between the two groups were not statistically significant. Additionally, the mean baseline 2 hGs were within the NGT range (<140 mg/dL).

Hazard ratios (HR) for the development of CFRD were derived from Cox proportional hazard models. The HR for every 10 mg/dL increase in baseline 1 hG glucose was 1.1 (95% CI 1.01, 1.2). The HR for every 10 mg/dL increase in baseline 2 hG glucose was 1.08 (95% CI 0.97, 1.21). From ROC calculations, we determined the baseline 1 hG that maximized the Youden index for detecting CFRD (optimizing sensitivity and specificity) was 141 mg/dL at 3 yrs (82% sensitivity, 36% specificity) and 153 mg/dL at 5 years (83% sensitivity and 56% specificity). The baseline 2 hG that maximized the Youden index for detecting CFRD was 102 mg/dL at both 3 yrs (71% sensitivity, 40% specificity) and 5 yrs (88% sensitivity and 49% specificity).

**Figure 2:**
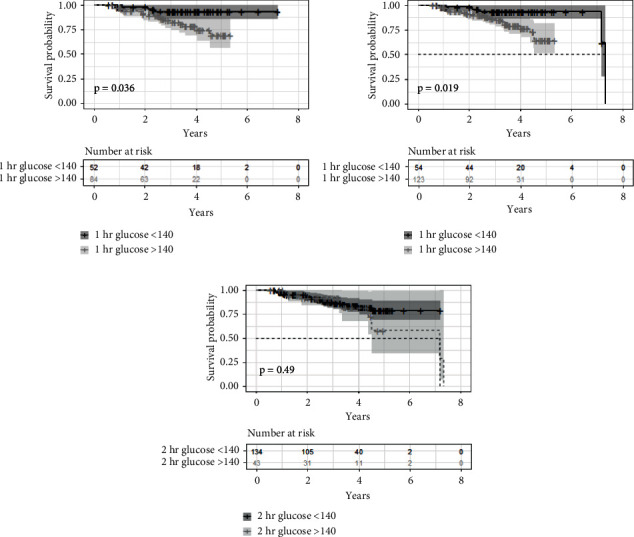
Kaplan–Meier survival curves comparing baseline 1 hG and the likelihood of developing diabetes between (a) patients with elevated 1 hG (≥140 mg/dL) with normal glucose tolerance (2 hG < 140 mg/dL) to low 1 hG (<140 mg/dL) with normal glucose tolerance, (b) patients with elevated 1 hG (≥140 mg/dL) including those with impaired glucose tolerance (2 hG ≥ 140 mg/dL) to low 1 hG (<140 mg/dL) including those with impaired glucose tolerance, and (c) patients with elevated 2 hG (≥140 mg/dL) to low 2 hG (<140 mg/dL), regardless of 1 hG concentrations. 1* *hG one-hour glucose and 2 hG two-hour glucose.

Kaplan–Meier analysis was performed in a subgroup of individuals with a normal baseline 2 hG ≤ 140 mg/dL to determine whether a high vs. low 1 hG predicted time to development of CFRD. First, the 1 hG cutpoint of 155 mg/dL was examined, as 1 hG concentrations at or above this threshold have been demonstrated to identify individuals at increased risk for type 2 diabetes. Among individuals with a normal 2 hG < 140 mg/dL, those with 1 hG ≥ 155 mg/dL were not more likely to develop CFRD over the next 5 years than individuals with 1 hG < 155 mg/dL (*p*=0.11). This analysis was repeated using a 1 hG cutpoint of 140 mg/dL. As shown in Figure 2(a), individuals with a normal 2 hG but 1 hG ≥ 140 mg/dL were more likely to develop CFRD over the next 5 years than individuals with 1 hG < 140 mg/dL (*p*=0.04). Interestingly, when this analysis was conducted with 2 hG alone, those with 2 hG under 140 mg/dL had a similar risk of developing CFRD compared with individuals who had a 2 hG ≥ 140 mg/dL (Figure 2(c)).

The values for 1 hG and 2 hG values in this CF population were well correlated (*R* = 0.73), but linear mixed effect modeling showed that the fitted line for each patient who developed CFRD crossed a 1 hG ≥ 155 mg/dL when 2 hG was >140 mg/dL, and crossed 2 hG of 140 mg/dL when 1 hG was >155 mg/dL. The average 1 hG was already 166 mg/dL when the 2 hG crossed 140 mg/dL, and the average 2 hG was only 132 mg/dL when the 1 hG crossed 155 mg/dL. Individuals with a 2 hG ≥ 200 mg/dL had an average 1 hG of 267 mg/dL. These results suggest the 1 hG threshold of 155 mg/dL was crossed earlier than the traditional 2 hG prediabetes cutpoint of 140 mg/dL; however, this analysis does not allow us to estimate the time interval between the crossing of events.

Last, we examined whether baseline 1 hG or 2 hG predicts BMI %ile and PFT trajectories in the overall cohort. Mixed-effect models demonstrated that baseline 1 hG did not predict trajectories of BMI% ile, FEV1% predicted, FVC% predicted, nor FEF 25–75%, even when adjusted for modulator use (*p* > 0.05). Baseline 2 hG predicted decline in FEV1% predicted (*p*=0.01), FVC% predicted (*p*=0.03), as well as FEF 25–75% predicted (*p*=0.001). These models retained significance when adjusted for CFTR modulator use.

## 4. Discussion

Our analysis of OGTTs in CF youth confirmed that elevations in 1 hG occur well before elevations in 2 hG. Elevations in 1 hG ≥ 140 mg/dL, despite normal fasting glucose and 2 hG, predicted progression to CFRD over the subsequent 5 years. However, while baseline 2 hG predicted declines in pulmonary function, 1 hG did not predict clinical declines. Therefore, our data suggest that 1 hG appears to be useful for predicting future CFRD risk and identifying individuals at high vs. low risk for developing diabetes, while 2 hG better predicted clinical declines. These findings highlight the unique strengths of measurements at each time point for informing clinical decision making.

Despite the clinical value of the OGTT, annual CFRD screening remains a significant challenge for many patients and CF centers. The US CF Annual Patient Registry demonstrates persistently low rates of screening OGTTs completed, with only 53% of youth and 24% of eligible adults with CF undergoing the recommended annual screening in 2020. Reasons for low OGTT screening rates include the perceived burdens of testing related to fasting, need for multiple blood draws, and the time and labor-intensive nature of the test [10–12]. Despite these challenges, latest CFRD guidelines have advocated for consideration of every 30-minute glucose sampling and OGTT screening as young as 6 years of age [4, 13]. Currently, collection of 1 hG during the OGTT is performed only in a minority of US CF centers [14], and notably, under current guidelines, the 1 hG concentrations do not alter clinical management. Although one reference has suggested more frequent screening for high-risk individuals with 1 hG > 200 mg/dL, as often as every 6 months [13], even annual collection of OGTTs remain challenging. Alternately, a more feasible and acceptable approach, supported by our findings, would be to use the 1 hG to identify individuals at low risk for CFRD who might benefit from reduced screening frequency.

Several studies in youth and adults with CF have highlighted the value of the 1 hG for predicting CFRD. In a 2013 longitudinal study from Germany including 385 pwCF >10 years of age with OGTT 1 hG concentrations, individuals with NGT + 1 hG > 200 mg/dL were at significantly increased risk for developing CFRD compared to those with NGT + 1 hG < 200 mg/dL (OR 2.81, 1.43–5.51) [8]. In a 2015 retrospective study of pediatric patients at a single US CF center, the authors described an increased 5-year risk of CFRD in youth with 1 hG > 160 mg/dL (OR 4.5, 1.7–18.7) [15]. Our group and others have previously described impairments in *β*-cell function in youth with CF at a 1 hG threshold of ≥155 mg/dL [16, 17]. More recently, with the aid of mathematical modeling, a cross-sectional study from Italy described impairments in *β*-cell function in children and adults with CF even with intermediate OGTT glucose elevations >140 mg/dL [18]. Our current study supports a 1 hG threshold >140 mg/dL for predicting increased CFRD risk, and it is likely that the degree of *β*-cell function impairment reflected by 1 hG elevations exists along a continuum, with higher 1 hG values above “normal” reflecting greater impairments.

When excluding individuals with impaired 2 hG, our survival curves detected an increased CFRD risk for those with a 1 hG > 140 mg/dl, but not >155 mg/dL. Thus it is possible that the 1 hG 155 mg/dL threshold may not be sensitive enough to distinguish individuals at lower vs. higher risk for progression to CFRD in the time period followed. If individuals with a 1 hG between 140 and 155 mg/dL are already exhibiting some degree of *β*-cell dysfunction, as has been previously described [18], these individuals may be confounding interpretation of results, and a threshold of 140 mg/dL may do a better job of identify those at lowest risk. Alternately, the lack of statistical significance may in part be due to the sample size and duration of follow-up limiting ability to detect this difference. Notably, when we treated 1 hG as a continuous variable, there was a significant increase in hazard for every 10 mg/dL increase. This implies that there actually is an increased risk for those with 1 hG > 155 mg/dL compared to 140 mg/dL, although we may not have had sufficient sample size to detect this when we dichotomized 1 hG, particularly when we limited analysis to those with a normal 2 hG.

While fasting and 2 hG values are commonly employed for diagnosing prediabetes and diabetes, there have an been increasing number of studies in people at risk for type 2 diabetes (T2D) linking 1 hG elevations ≥155 mg/dL with early cardiometabolic disease in addition to increased risk for T2D [19, 20]. Although the pathophysiology of T2D and CFRD differ, current OGTT cut points for diagnosing diabetes based on the 2 hG in the two populations are the same. While OGTT elevations ≥200 mg/dL at intermediate timepoints between 0 and 2 h are currently considered “indeterminate” in pwCF, at least one cross-sectional study in youth demonstrated that early rises in 1 hG, but not 2 hG, may be associated with lower pulmonary function [21]. However, subsequent longitudinal studies from this group did not identify an association between 1 hG elevations and changes in FEV1 over time or increased pulmonary exacerbation risk [15]. In adults with CF, a 2016 study from Canada including OGTT 1 hG concentrations identified a negative association between 1 hG and FEV1 [22]. However, a larger 2021 study including adults with CF from both Canada and France did not identify any associations between 1 hG and lower lung function nor BMI over a 4-year follow-up [23]. Interestingly, a 2018 study from France examining 1 hG and 2 hG trajectories, incorporating longitudinal OGTT data, found no relationship in children between either 1 hG or 2 hG trajectories and rate of FEV1 decline, while in contrast, in adults, higher and increasing 2 hG trajectories were associated with greater FEV1 decline [24].

Notably, CF therapies over the past several decades have continued to improve, and the latest highly effective CFTR modulator therapies (HEMT) are changing the landscape of CF such that previously identified relationships between dysglycemia and pulmonary or nutritional outcomes may change. Importantly, early glycemic abnormalities predict nutritional and pulmonary function decline, and might warrant earlier intervention, particularly as individuals with CF are becoming relatively healthier compared to previous decades, warrants ongoing study in the HEMT era.

Whether the 1 hG might be able to replace the 2 hG has also been a growing area of interest in type 1 diabetes [10] and T2D screening. A study by Ha and colleagues in Pima Indians, a group at very high risk for T2D and the original group in which OGTT prediabetes and diabetes criteria were established, demonstrated that 1 hG is highly correlated with 2 hG (*R*^2^ = 0.89) [25]. Their findings suggest that 1 hG could serve as an accurate, alternate screening test for prediabetes and diabetes in populations at risk for T2D. We examined the relationship between 1 hG and 2 hG in our CF population and found; however, that this correlation was lower than that previously described in the Pima Indians. We speculate that reasons behind these differences in correlation between 1 hG and 2 hG in the two populations may be underlying diabetes pathophysiology. Gradually progressive insulin resistance and eventual *β*-cell failure, as seen in T2D, appear to impact OGTT glucose patterns differently than in disease states where insulin insufficiency is the primary defect, as seen in early CFRD. Additionally, fluctuations in insulin resistance and inflammation from intermittent CF pulmonary exacerbations, as well as the insulin resistance of puberty in this largely adolescent cohort, may have influenced the relationships observed over time between 1 hG and 2 hG in our population.

Furthermore, our data demonstrate that a higher 1 hG rise is seen in CF when compared to previously described 1 hG elevations in individuals at risk for T2DM [26]. Our model, which shows that pwCF develop significant elevations in 1 hG prior to 2 hG, as well as current knowledge of the pathophysiology of CF, support the hypothesis that rises in 1 hG occur because of *β*-cell dysfunction. It has been well described that delayed insulin secretion, as detected during an OGTT, is common in CF and is attributed to early loss of the first-phase insulin response. While this loss of first-phase insulin response is not unique to CF and has also been described in T2D [27, 28], it remains a prominent contributor to the pathophysiology of CFRD and these early abnormalities in insulin secretion may be more readily detected by a 1 hG [3, 17]. Other studies have described greater associations between 1 hG and loss of first phase insulin than 2 hG, although both are associated with reduced insulin secretion and insulin resistance [29].

Our study has several strengths and limitations. Strengths include examination of both the 1 hG and 2 hG and their ability to predict CFRD as well as pulmonary function and BMI outcomes in a longitudinal cohort of CF youth. We are aware of 4 other longitudinal studies that have examined a similar question [8, 15, 23, 24], with only 3 including pediatric CF patients. We examined a lower 1 hG threshold than is typically assessed and found that a 1 hG > 140 mg/dL, despite a normal 2 hG, is associated with increased CFRD risk. An important take-away is that individuals with a low 1 hG < 140 mg/dL may warrant less frequent OGTT screening, for example, every 3–5 years rather than annually, which would reduce both patient burden and healthcare costs. Limitations include data capture from a single-center and the retrospective study design. We examined time to CFRD diagnosis based on OGTT results; however, we did not capture CFRD diagnoses by other methods including hyperglycemia during hospitalizations or other indications for insulin start. Additionally, this study was conducted in youth, and there may be a confounding effect of the insulin resistance associated with puberty on the development of dysglycemia. Furthermore, our data were examined prior to the widespread adoption of the latest triple combination CFTR modulator, elexacaftor/tezacaftor/ivacaftor, Food and Drug Administration (FDA) approved in October 2019 [1]. At least one study has demonstrated decreased incidence of CFRD over 5 years in individuals treated with the highly effective CFTR modulator ivacaftor [30]. On the other hand, evidence also suggests that rising BMI will increase insulin resistance, and over time, this may increase the risk for diabetes in a population with underlying *β*-cell dysfunction. Thus, the long-term impacts of CFTR modulator therapy on the pathophysiology and progression of CFRD remain to be seen. Prospective, multicenter studies to determine the clinical implications of early glycemic abnormalities and the role of early intervention targeting insulin deficiency and dysglycemia, along with studies investigating strategies to reduce the burden of diabetes screening in the HEMT era, are now needed.

## Figures and Tables

**Table 1:** Baseline demographics for the cross-sectional and longitudinal cohorts.

	Group 1 (cross-sectional cohort)	Group 2 (longitudinal cohort)
	*n* = 243	*n* = 177
Male, *n* (%)	120 (49%)	94 (53%)
Race		
Caucasian, *n* (%)	194 (80%)	142 (80%)
Other, *n* (%)	49 (20%)	35 (20%)
Age (years)	12.66 ± 3.03	12.42 ± 2.58
Fasting OGTT (mg/dL)	89 ± 8	89 ± 8
1-hour glucose (mg/dL)	163 ± 45	162 ± 45
2-hour glucose (mg/dL)	117 ± 33	116 ± 33
BMI%ile	18.77 ± 2.95	19.01 ± 3.05
HbA1c (%)	5.5 ± 0.4	5.5 ± 0.4
Genotype, *n* (%)		
Residual function mutations	22 (9%)	14 (8%)
2 minimal function mutations	213 (88%)	159 (90%)
Unknown	8 (3%)	4 (2%)
FEV1% predicted	91.64 ± 16.88	90.69 ± 15.71
FVC% predicted	99.13 ± 14.98	98.29 ± 13.67
FEV1/FVC	0.92 ± 0.09	0.92 ± 0.08
CFTR modulator use at baseline, *n* (%)		
Yes	42 (17%)	21 (12%)
No	201 (83%)	156 (88%)
Started on modulator during follow-up, *n* (%)		
Yes	90 (37%)	79 (45%)
No	153 (63%)	98 (55%)

**Table 2:** Baseline characteristics of patients who did or did not develop CFRD during study period.

	CF without diabetes	CFRD progressors	*p* value
	*n* = 149	*n* = 28	
Male, *n* (%)	82 (55%)	12 (43%)	0.33
Race, *n* (%)			0.75
Caucasian	119 (80%)	23 (82%)	
Other	30 (20%)	5 (18%)	
Age (years)	12.53 ± 2.68	11.85 ± 1.91	0.20
Fasting OGTT (mg/dL)	90 ± 8	89 ± 9	0.49
1-hour glucose (mg/dL)	160 ± 44	173 ± 48	0.16
2-hour glucose (mg/dL)	114 ± 32	127 ± 39	0.07
BMI% ile	19.26 ± 3.19	17.59 ± 1.57	0.09
HbA1c (%)	5.5 ± 0.4	5.6 ± 0.3	0.19
Genotype, *n* (%)			0.15
Residual function mutations	14 (9%)	0 (0%)	
2 minimal function mutations	131 (88%)	28 (100%)	
Unknown	4 (3%)	0 (0%)	
FEV1% predicted	92.29 ± 16.14	81.69 ± 9.31	0.08
FVC% predicted	99.38 ± 14.28	92.13 ± 7.43	0.17
FEV1/FVC	0.93 ± 0.08	0.89 ± 0.08	0.23
Time between 1^st^ and last visit (years)	3.24 ± 1.34	2.75 ± 1.73	0.09
Age at CFRD diagnosis (years)	—	15.20 ± 2.57	

## Data Availability

The data used to support the findings of this study are available from the corresponding author upon request.

## Abbreviations:

1 hG: 1-hour glucose
2 hG: 2-hour glucose
BMI: Body mass index
CFRD: Cystic fibrosis-related diabetes
CFTR: Cystic fibrosis transmembrane conductance regulator
FDA: Food and Drug Administration
FVC: Forced vital capacity
A1c: Hemoglobin A1c
HEMT: Highly effective CFTR modulator therapies
NGT: Normal glucose tolerance
OGTT: Oral glucose tolerance testing
PwCF: People with cystic fibrosis
PFT: Pulmonary function testing
T1D: Type 1 diabetes
T2D: Type 2 diabetes.
